# Modeling Catecholaminergic Polymorphic Ventricular Tachycardia using Induced Pluripotent Stem Cell-derived Cardiomyocytes

**DOI:** 10.5041/RMMJ.10086

**Published:** 2012-07-31

**Authors:** Atara Novak, Lili Barad, Avraham Lorber, Joseph Itskovitz-Eldor, Ofer Binah

**Affiliations:** 1 The Sohnis Family Stem Cells Center, Technion - Israel Institute of Technology, Haifa, Israel;; 2 The Rappaport Family Institute for Research in the Medical Sciences, Technion - Israel Institute of Technology, Haifa, Israel;; 3 Ruth & Bruce Rappaport Faculty of Medicine, Technion - Israel Institute of Technology, Haifa, Israel;; 4 Department of Pediatric Cardiology, Rambam Health Care Campus, Haifa, Israel; and; 5 Department of Obstetrics and Gynecology, Rambam Health Care Campus, Haifa, Israel

**Keywords:** Adrenergic stimulation, cardiac myocytes, catecholaminergic polymorphic ventricular tachycardia (CPVT), delayed afterdepolarizations, induced pluripotent stem cells (iPSC), inherited arrhythmias

## Abstract

Catecholaminergic polymorphic ventricular tachycardia (CPVT) is an inherited arrhythmogenic cardiac disorder characterized by life-threatening arrhythmias induced by physical or emotional stress, in the absence structural heart abnormalities. The arrhythmias may cause syncope or degenerate into cardiac arrest and sudden death which usually occurs during childhood. Recent studies have shown that CPVT is caused by mutations in the cardiac ryanodine receptor type 2 (RyR2) or calsequestrin 2 (CASQ2) genes. Both proteins are key contributors to the intracellular Ca^2+^ handling process and play a pivotal role in Ca^2+^ release from the sarcoplasmic reticulum to the cytosol during systole. Although the molecular pathogenesis of CPVT is not entirely clear, it was suggested that the CPVT mutations promote excessive sarcoplasmic reticulum Ca^2+^ leak, which initiates delayed afterdepolarizations (DADs) and triggered arrhythmias in cardiac myocytes. The recent breakthrough discovery of induced pluripotent stem cells (iPSC) generated from somatic cells (e.g. fibroblasts, keratinocytes) now enables researches to investigate mutated cardiomyocytes generated from the patient’s iPSC. To this end, in the present article we review recent studies on CPVT iPSC-derived cardiomyocytes, thus demonstrating in the mutated cells catecholamine-induced DADs and triggered arrhythmias.

## BACKGROUND

Sudden cardiac death caused by a ventricular arrhythmia is a disastrous event, especially when it occurs in young individuals. Among the five major arrhythmogenic disorders occurring in the absence of structural heart diseases is catecholaminergic polymorphic ventricular tachycardia (CPVT), which is a highly lethal form of inherited arrhythmogenic disease characterized by adrenergically mediated polymorphic ventricular tachycardia.^1^–^4^ In response to physical activity or emotional stress, this disease is characterized by episodes of syncope, seizures, or sudden death.^1^,^2^ CPVT was first described as a case report by Reid et al.^5^ who reported on a bidirectional ventricular tachycardia triggered by physical effort and emotional stress in a 6-year-old girl (who survived a cardiac arrest) with no evidence of any structural heart disease. Later on, in 1978 and 1995, Coumel et al.^6^ and Leenhardt et al.^7^ reported a series of cases in familial as well as in sporadic forms of the arrhythmia and introduced the term catecholaminergic polymorphic ventricular tachycardia (CPVT) to refer to a disease characterized by adrenergically mediated bidirectional and/or polymorphic ventricular tachycardia in the absence of cardiac pathology. In 2001, Priori et al.^8^ and Lahat et al.^9^ identified mutations in the cardiac ryanodine receptor (RyR2) and cardiac calsequestrin (CASQ2) genes, respectively, underlying autosomal dominant and autosomal recessive forms of the disease. RyR2 is a cardiac Ca^2+^ release channel located on the sarcoplasmic reticulum (SR) membrane and has a key role in the process of calcium-induced calcium release (CICR) during the excitation–contraction (E–C) coupling.^10^,^11^ CASQ2 is a high-capacity, low-affinity Ca^2+^-binding protein, operating as a major Ca^2+^-buffering factor, and together with RyR2 forms the SR Ca^2+^ release unit.^8^,^10^,^11^ Since both proteins are key players in the E–C coupling process, the functional derangements in intracellular Ca^2+^-handling resulting from the mutated RyR2 and CASQ2 genes may cause delayed afterdepolarizations (DADs), which constitute the major electrophysiological mechanism underlying CPVT.^2^,^12^ The prevalence of CPVT in the population is not completely known and has been estimated as 1:10,000.^4^ If left untreated, 80% of CPVT patients will develop symptoms (ventricular tachycardia, ventricular fibrillation, syncope, sudden death) by the age of 40, with overall mortality of 30%–50%.^13^ Because β-adrenergic blockers and implantable cardioverter defibrillator (ICD) therapy can rescue most CPVT cases, early diagnosis by means of clinical evaluations and genetic screening is possible and crucial. Hence, ICD may be considered for primary prevention of a cardiac arrest in CPVT patients in whom severe ventricular arrhythmias or recurrent syncope are observed in the presence of β-adrenergic blocking therapy.^2^

In general, two genetic variants of CPVT have been identified. One is transmitted as an autosomal dominant trait caused by mutations in the gene encoding RyR2 (CPVT1) which is responsible for 50%–55% of all CPVT patients. Presently, more than 150 mutations have been identified in the RyR2 gene,^14^ preferentially located in four highly conserved regions (domains I–IV) of the gene.^15^ The second variant is an autosomal recessive form caused by mutations in the cardiac specific isoform of the calsequestrin gene CASQ2 (CPVT2) which represents only 3%–5% of CPVT patients.^4^ To date, 15 CASQ2 mutations have been identified in the short arm of chromosome 1 which lead to severe decrease or complete loss of the CASQ2 protein.^4^

## THE MOLECULAR MECHANISM UNDERLYING CPVT

### Key Elements of the Excitation–Contraction Coupling Machinery

The delicate balance that regulates Ca^2+^ fluxes between the intracellular compartment and the extracellular space in cardiomyocytes is critical to ensure cellular viability, preserve normal contractile function, and to provide a stable heart rhythm.^2^,^16^ During the plateau phase of the cardiac action potential, a small amount of Ca^2+^ enters the cardiomyocytes through the voltage-dependent L-type Ca^2+^ channels, causing Ca^2+^ release into the cytosol through the RyR2 channel located in the SR membrane. This process of CICR is the basis of cardiac E–C coupling.^11^,^16^,^17^ The attainment of higher concentrations of cytosolic Ca^2+^ causes activation of the contractile filaments of the cardiac sarcomere, which is followed by diminution of Ca^2+^ concentration to the diastolic level, thus causing relaxation. Ca^2+^ levels are lowered to diastolic values by means of three systems: 1) the sarcoplasmic endoplasmic reticulum calcium (SERCA2, the cardiac SERCA) which is responsible for reuptake of ∼75% of Ca^2+^ into the SR; 2) The sodium–calcium exchanger (NCX) which extrudes the remaining portion of cytosolic Ca^2+^ from the cytoplasm; and 3) a small portion of the Ca^2+^ is extruded to the extracellular space by means of an ATP-operated Ca^2+^ pump.^11^

### The Ca^2+^ Release Unit (CRU)

RyR2 is a large homotetrameric Ca^2+^ release channel located on the SR membrane. The RyR2 channels are composed of four pore-forming monomers, comprising a relatively small C-terminal transmembrane domain and a large N-terminal domain that protrudes into the cytosol. The cytoplasmic domain of RyR2 is stabilized by FKBP12.6 and is essential for channel closure during diastole.^8^,^17^,^18^ The CASQ2 is the major Ca^2+^ storage protein in the SR and is capable of binding luminal Ca^2+^ (40–50 Ca^2+^ ions/molecule) during diastole in order to prevent Ca^2+^ precipitation and to reduce the ionic Ca^2+^ concentration.^19^ On the luminal side, RyR2 binds junctin and triadin, which anchor the Ca^2+^-buffering protein CASQ2,^11^ collectively forming the SR Ca^2+^ release unit (CRU). The CRU is responsible for SR Ca^2+^ release, which is triggered by increased cytosolic Ca^2+^ resulting from opening of the L-type channel (CICR).^11^ In addition, CASQ2 has been suggested to modulate the activity of RyR2 directly.^20^ Under adrenergic stimulation, β-adrenergic receptors activate a GTP-binding protein that stimulates adenylyl cyclase to produce cAMP, which in turn activates protein kinase A (PKA). This kinase phosphorylates RyR2 and other central proteins related to E–C coupling, such as phospholamban and the L-type Ca^2+^ channels, thus causing gain of function of Ca^2+^ cycling in cardiomyocytes in response to adrenergic activation. FKBP12.6 stabilizes RyR2 in the closed state, and the hyperphosphorylation of RyR2 by PKA causes FKBP12.6 dissociation from RyR2, thereby increasing the open probability of RyR2.^21^ Moreover, adrenergic stimulation also increases the activity of the SERCA pump via the phosphorylation of phospholamban by PKA which stabilizes SERCA.

### The mechanism of CPVT

**Figure 1:**
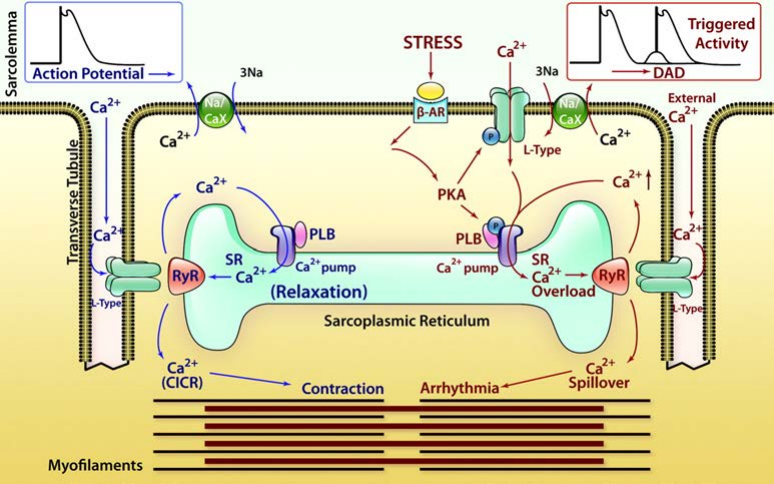
**Ca^2+^-induced Ca^2+^ release (CICR), store overload-induced Ca^2+^ release (SOICR), and triggered arrhythmia.** The left part of the diagram (in blue) depicts the mechanism of CICR, in which an action potential activates the voltage-dependent L-type Ca^2+^ channel, leading to a small Ca^2+^ influx. This Ca^2+^ entry opens the RyR2 channel in the sarcoplasmic reticulum (SR), resulting in SR Ca^2+^ release and muscle contraction. The right part of the diagram (in red) denotes the mechanism of SOICR, in which spontaneous SR Ca^2+^ release or Ca^2+^ spillover occurs under conditions of SR Ca^2+^ overload caused, for example, by stress via the β-adrenergic receptor (b-AR)/protein kinase A (PKA)/phospholamban (PLB) signaling pathway. SOICR can activate the Na^+^/Ca^2+^ exchanger (Na/CaX), which, in turn, can lead to delayed afterdepolarizations (DADs) and triggered activities. Reprinted from Priori and Chen^15^ with permission.

*In-vitro* studies suggested that the RyR2 and CASQ2 mutations cause the CRU to open spontaneously without being triggered by voltage-gated Ca^2+^ influx, thereby leading to intracellular Ca^2+^ overload.^1^,^2^ Increased intracellular Ca^2+^ can trigger early or delayed afterdepolarizations (oscillations of the membrane potential that occur during the plateau/ repolarization phase of the action potential or after its completion, respectively) that can reach the threshold potential and cause triggered activity.^15^ Intracellular Ca^2+^ overload leads to NCX activation which extrudes Ca^2+^ in exchange for Na^+^ with a stoichiometry of 1:3, thereby generating a net inward current (the so-called transient inward current, I_Ti_).^2^ The transient inward current induces DADs which may reach threshold and trigger premature ventricular beats and ventricular arrhythmias (demonstrated in Figure 1) by a mechanism called triggered activity.^2^

## EXPERIMENTAL MODELS OF CPVT

**Figure 2:**
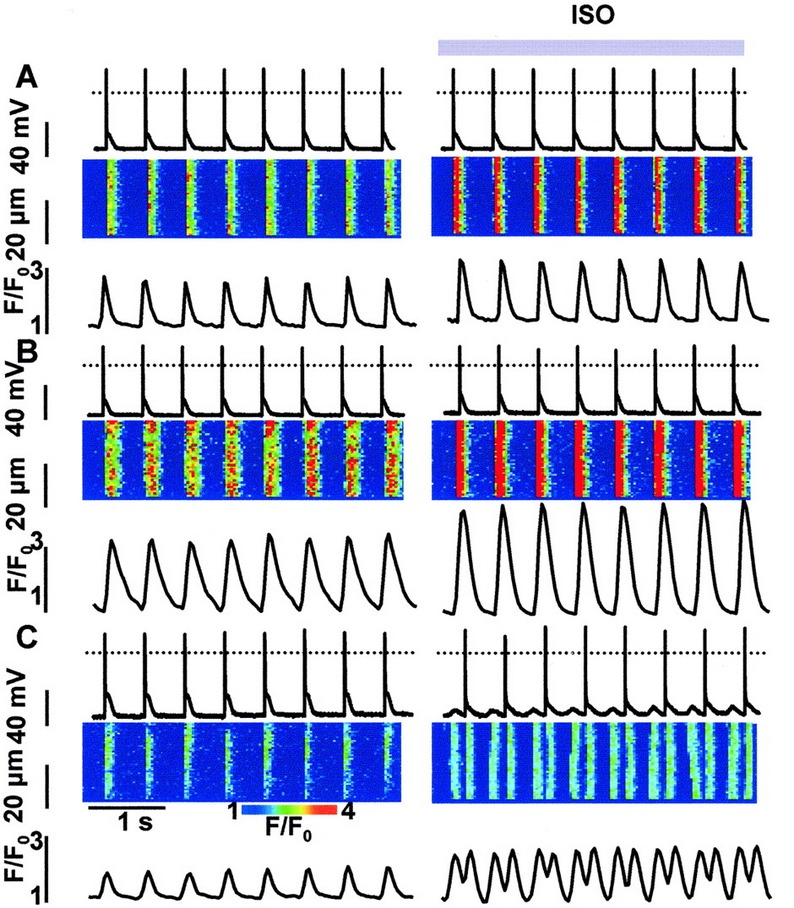
**Disturbances in rhythmic [Ca^2+^]_i_ transients and membrane potential induced by ISO in myocytes expressing CASQ2^D307H^.** Recordings of membrane potential (upper traces), along with line scan images (middle) and time-dependent profiles of [Ca^2+^]_i_ (lower traces) in myocytes infected with *Ad*-*Control* **(A)**, *Ad-CASQ2^WT^* **(B)**, and *Ad-CASQ2^D307H^* **(C)** vectors before and after exposure of the myocytes to 1 μmol/L ISO. Myocytes were stimulated at 2 Hz in the current-clamp mode. Reprinted from Viatchenko-Karpinski et al.^12^ with permission.

The electrophysiological mechanisms of CPVT, which result from mutations in the RyR2 and CASQ2 genes, were investigated in several *in-vitro* and *in-vivo* models.^12^,^22^–^24^ The first CPVT transgenic mouse model was produced by Priori’s group in 2005. These authors introduced the RyR2 R4496C mutation into the mouse genome by homologous recombination and successfully reproduced the human phenotype.^24^ This *in-vivo* model proved the concept that RyR2 mutations may cause polymorphic and bidirectional ventricular tachycardia in a knock-in mouse model and mimic the clinical phenotype of CPVT patients.^24^ Subsequently, Priori’s group demonstrated that myocytes isolated from the heart of the RyR2 R4496C knock-in mouse generated DADs and triggered activity upon exposure to β-adrenergic stimulation, while the control myocytes isolated from wild-type mouse did not.^25^ Similar results were reported by Kannankeril et al. who generated a knock-in mouse model of RyR2 R176Q mutation and demonstrated the disease phenotype *in vivo* and *in vitro*.^26^ Priori’s group was also the first to generate *in-vitro* modeling of the CASQ2 D307H mutation using infected rat myocytes with adenoviruses engineered to express the mutated CASQ2. In this study the authors demonstrated that the mutated myocytes developed DADs upon adrenergic stimulation while the control myocytes did not, as shown in Figure 2. This model provided the first evidence that mutant CASQ2 can generate DADs.^12^ Using the same model of rat myocytes infected with adenoviruses, Terentyev et al. and di Barletta el al. generated *in-vitro* models of the CASQ2 R33Q and L167H mutations, respectively, and reported on the generation of DADs in the mutated myocytes.^22^,^23^ Nonetheless, these models suffer from several limitations because the myocytes express both the mutant and the endogenous wild-type CASQ2. In 2006, Knollmann et al. generated CASQ2-null (CASQ2^−/−^) mice which were viable and exhibited the human-like arrhythmias. Despite the absence of CASQ2, these animals maintained relatively normal Ca^2+^ release and contractile function. However, myocytes isolated from these mice had exceptional increases in SR volume, increased gain of Ca^2+^-induced SR Ca^2+^ release, and increased diastolic SR Ca^2+^ leak.^27^ Other transgenic models with deficient RyR2 or CASQ2 genes were generated and have confirmed the correlation between the arrhythmogenic phenotype to the mutant genes, although the observed phenotype did not always resemble the human disease.^28^

## HUMAN MODELS OF CPVT

**Figure 3:**
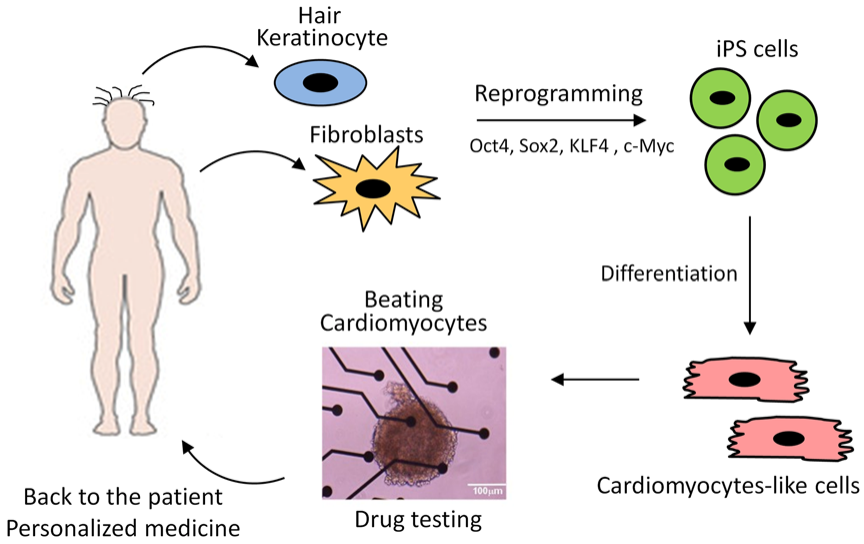
**Induced pluripotent stem cells—derivation and applications.** Induced pluripotent stem cells (iPSC) can be generated from adult cells as hair keratinocytes and skin fibroblasts by introducing specific genes. The cells are reprogrammed and become pluripotent, resembling embryonic stem cells. Among others, iPSC can be differentiated into beating cardiomyocytes and used as disease models, for drug testing, and in the future for transplantation in cell replacement therapy.

**Figure 4:**
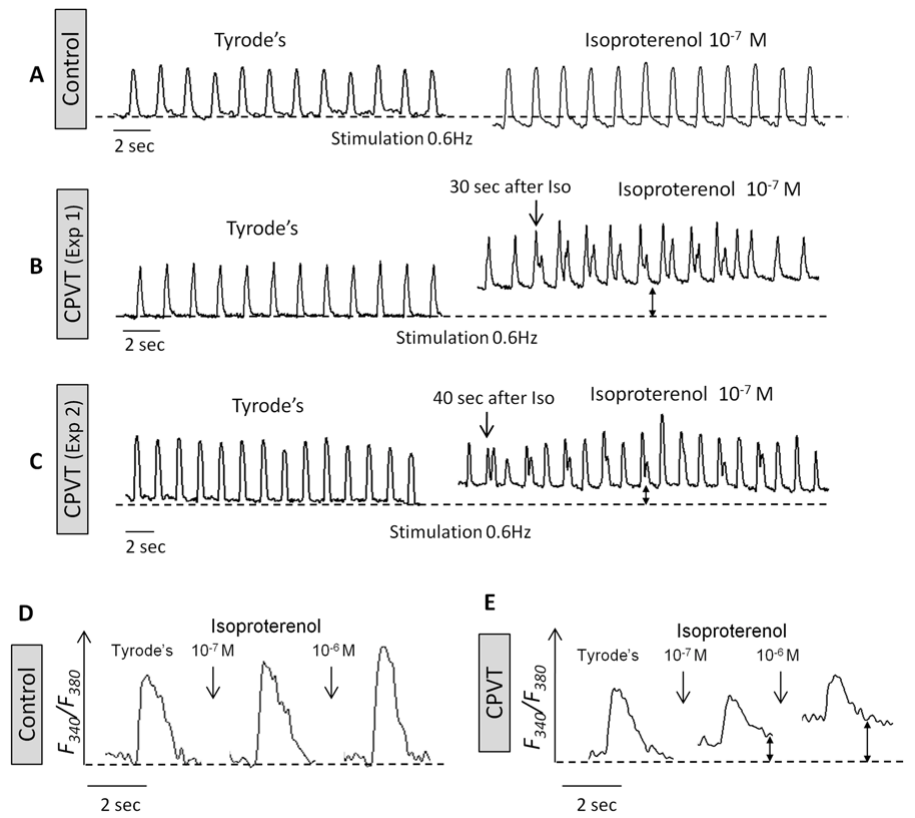
**The effects of isoproterenol on the [Ca^2+^]_i_ transients and contractions in control and CPVT iPSC cardiomyocytes.** **(A)** Representative contraction tracings of control iPSC cardiomyocytes (43-day-old embryoid body (EB)) stimulated at 0.6 Hz, in the absence (Tyrode’s) and presence of isoproterenol. **(B,C)** Representative contraction tracings of CPVT iPSC cardiomyocytes (33- and 38-day-old EBs, respectively) stimulated at 0.6 Hz, in the absence (Tyrode’s) and the presence of isoproterenol. Note that after-contractions developed only in the CPVT cardiomyocytes in the presence of isoproterenol. **(D,E)** Representative [Ca^2+^]_i_ transients of control iPSC cardiomyocytes (40-day-old EB) and CPVT iPSC cardiomyocytes (34-day-old EB), respectively, before and 5 minutes after isoproterenol perfusion. Reprinted from Novak et al.^37^ with permission.

**Figure 5:**
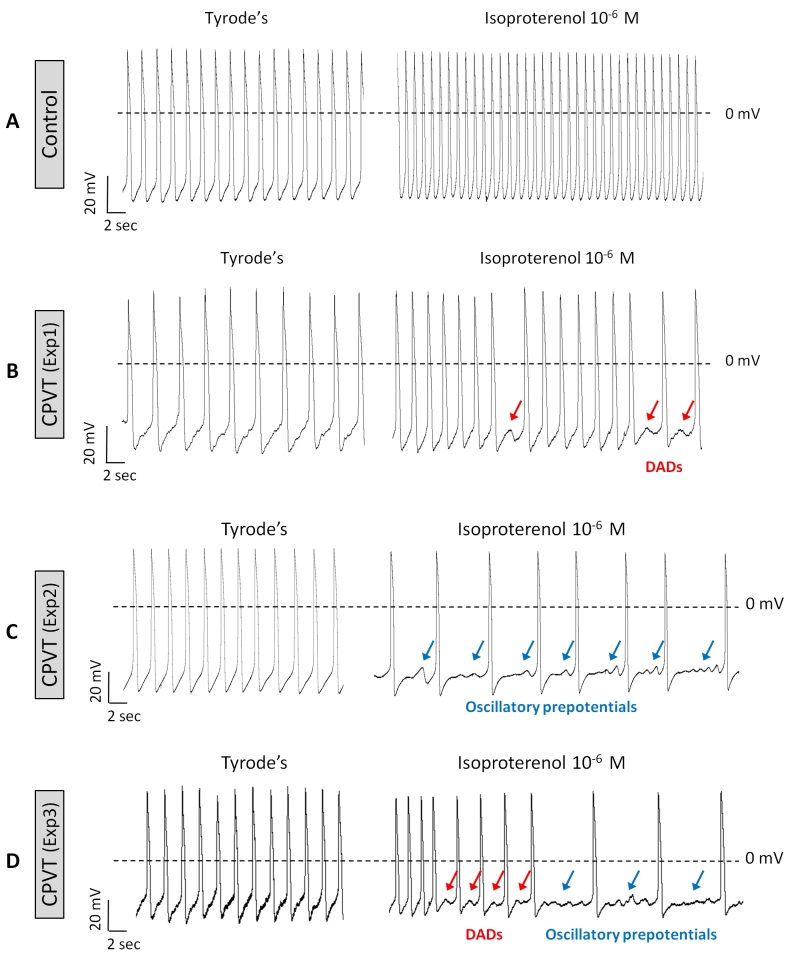
**DADs and oscillatory prepotentials in CPVT iPSC cardiomyocytes induced by isoproterenol.** (**A**) Representative spontaneous recordings from control cardiomyocytes (30-day-old embryoid body (EB)), and (**B**,**D**) CPVT cardiomyocytes (32-, 30-, and 32-day-old EBs, respectively) in the absence (Tyrode’s) and presence of isoproterenol. Note that only in CPVT cardiomyocytes, isoproterenol caused DADs (red arrows) and negative chronotropic effect. Reprinted from Novak et al.^37^ with permission.

The recent discovery of genomic reprogramming of human somatic cells into induced pluripotent stem cells (iPSC) offers an innovative and practical approach to study inherited diseases including heart pathologies. Induced pluripotent stem cells are the product of somatic cell reprogramming to an embryonic-like state. Human iPSC have been generated from various types of somatic cells, most commonly skin fibroblasts, but also from hair keratinocytes isolated from plucked human hair which offers significant advantages over isolating skin fibroblasts through an invasive surgical procedure.^29^ This process of reprogramming occurs by the introduction of a defined and limited set of transcription factors and by culturing these cells under embryonic stem cell conditions. Somatic cell reprogramming by the induction of four ectopic genes (OCT3/4, SOX2, C-MYC, and KLF4) by retroviral insertion was first described in mouse cells,^30^ and later on in human cells,^31^ by Yamanaka’s group. This new technology allows us to investigate cardiac disorders *in vitro* and opens new opportunities for investigating the diseases’ mechanism *in vitro*, developing new drugs, predicting their toxicity, and optimizing current treatment strategies (Figure 3). Recently, several groups reported on the generation of patient-specific iPSC of the inherited arrhythmias—LEOPARD syndrome,^32^ long-QT1,^33^ long-QT2,^34^,^35^ and Timothy syndrome^36^—and demonstrated the capacity of these cells to give rise to functional cardiomyocytes that display the electrophysiological characteristics of the disorder. In the last year, three groups reported on the generation of iPSC-derived cardiomyocytes from CPVT2^37^ and CPVT1^38^,^39^ patients. In this regard, our group was the first to generate cardiomyocytes from CPVT patients carrying the missense D307H in the CASQ2 gene, which in response to β-adrenergic stimulation generated DADs and triggered activity.^37^ Specifically, Novak et al. reported on the generation of cardiomyocytes from two CPVT2 patients: a 12-year-old boy and a 30-year-old woman carrying the missense mutation D307H in the CASQ2 gene, which exhibited the key features of catecholaminergic-mediated arrhythmogenesis.^37^ The D307H mutation (associated with a change from a negatively charged aspartic acid to a positively charged histidine) causes reduced affinity of the mutant CASQ2 to Ca^2+^, thereby resulting in Ca^2+^ spillover during adrenergic stress.^4^ To decipher the cellular mechanisms of CPVT we performed patch clamp and intracellular Ca^2+^ and contraction measurements from iPSC cardiomyocytes generated from healthy and diseased individuals. In agreement with previous studies reporting a lower resting heart rate among CPVT patients,^40^,^41^ we found that the mean spontaneous beating rate of CPVT iPSC cardiomyocytes was slower by 34% than control cardiomyocytes. These findings still need to be established in a larger number of cells from different clones. The adrenergically mediated arrhythmogenic features of CPVT2 cardiomyocytes were demonstrated by exposing the cells to the β-adrenergic agonist isoproterenol. While isoproterenol or pacing were not arrhythmogenic in control cardiomyocytes, in 47% of the CPVT cardiomyocytes isoproterenol caused the generation of after-contractions (which are the mechanical equivalent of DADs) and triggered beats (Figure 4B and C), and in 33% of mutated cardiomyocytes pacing alone caused after-contractions in the absence of isoproterenol. Further, in contrast to control cardiomyocytes in which isoproterenol commonly causes positive inotropic and lusitropic (increased rate of relaxation) effects, CPVT cardiomyocytes were unresponsive. Further, in CPVT (but not in control) cardiomyocytes, isoproterenol caused marked elevation in the resting tension level, probably resulting from a prominent diastolic [Ca^2+^]_i_ rise (Figure 4E). This last-mentioned phenomenon is the principal feature of the CASQ2 mutation, as previously demonstrated in paced cardiomyocytes derived from a CASQ2-deficient mutant mouse.^42^ Next, by means of the patch clamp technique, we^37^ demonstrated (Figure 5) for the first time that, in addition to the familiar DADs occurring in CPVT2-mutant cardiomyocytes in response to β-adrenergic stimulation,^8^,^12^,^43^ isoproterenol also caused arrhythmogenic depolarizing oscillatory prepotentials, which were originally described in cardiac muscle by Bozler in 1942.^44^,^45^ In contrast to DADs which follow the action potential and therefore appear during the *early* diastolic depolarization, oscillatory prepotentials are defined as diastolic voltage oscillations which appear during the *late* diastolic depolarization.^46^,^47^ The oscillatory prepotentials have longer duration than DADs, they overshoot and undershoot the late diastolic depolarization, and they grow progressively in amplitude until reaching the threshold and initiating spontaneous activity.^46^,^47^ Further, 75% of the CPVT cardiomyocytes exposed to isoproterenol developed DADs (Figure 5B), oscillatory prepotentials (Figure 5C), or both (Figure 5D). Finally, ultrastructural analysis of CPVT2 cardiomyocytes showed a small expansion of the SR cisternae,^37^ as previously reported in a mouse model of CPVT with a deficient cardiac CASQ2.^27^,^43^,^48^ This expansion was suggested to constitute a compensatory response for the loss of SR Ca^2+^ buffering by the mutated CASQ2.^27^

**Figure 6:**
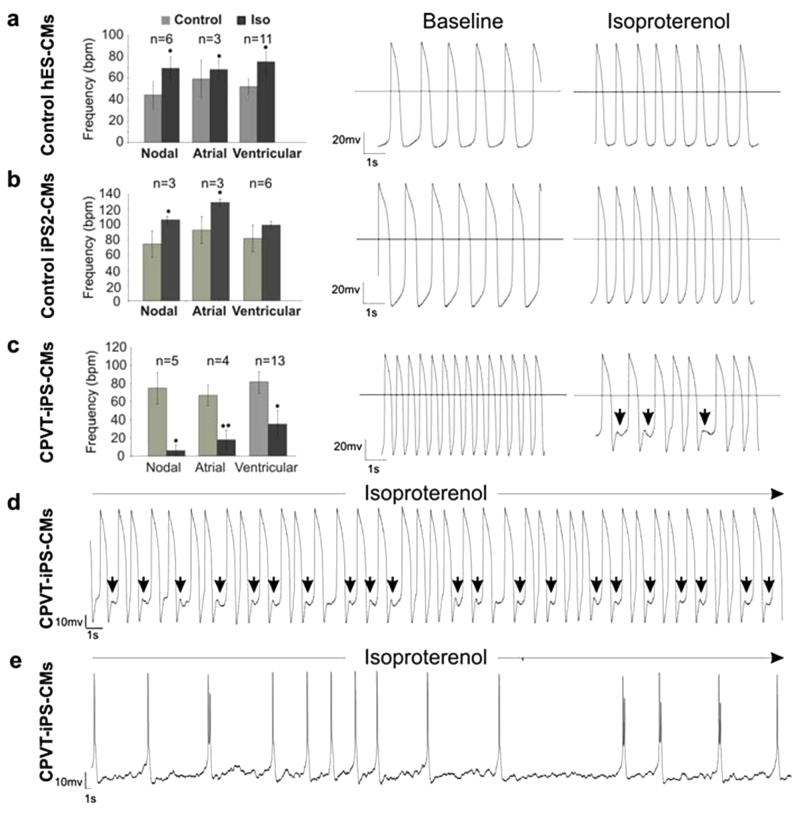
**Electrophysiological characterization of control and CPVT1 cardiomyocytes (CM).** Action potentials in single cardiomyocytes were recorded by the whole-cell patch clamp method in the current-clamp mode, and the beating frequency of cells at basal state and after 1 μM isoproterenol (Iso) treatment was determined. Cardiomyocytes differentiated from control embryonic stem cells **(A)** and iPSC lines **(B)** responded to 1 μM isoproterenol stimulation with positive chronotropy and did not show any arrhythmia after treatment. Representative traces of action potentials before and after isoproterenol treatment are shown in the middle and right panels, respectively. **(C)** A large fraction of cardiomyocytes (22 out of 38 cells, 57.9%) derived from CPVT1 iPSC responded to isoproterenol with negative chronotropy (left panel), and 13 out of 38 (34.2%) CPVT1 cardiomyocytes exhibited arrhythmia and DADs (arrows above traces in the right panel). **(D,E)** Representative traces of arrhythmic action potentials in two additional cells exposed to isoproterenol. The action potential traces in the cell depicted in panel **(D)** show putative DADs (arrows). The *n* values in panels **(A,C)** show the total number of analyzed cells. Error bars show SEM. ^*^P < 0.05, ^**^P < 0.01. Reprinted from Fatima A. et al.^38^ with permission of S. Karger AG, Medical and Scientific Publishers, Basel, Switzerland.

More recently, Fatima and co-workers generated CPVT1-specific iPSC from a 46-year-old woman diagnosed with CPVT1, who carried the novel heterozygous autosomal dominant missense mutation F2483I in the RYR2 gene. This mutation is localized in the FKBP12.6-binding domain of the RYR2 protein.^38^ In agreement with our findings,^37^ these authors showed that while in all control cardiomyocytes isoproterenol caused a positive chronotropic effect, but no arrhythmias, in 57.9% of the CPVT cardiomyocytes isoproterenol caused a negative chronotropic effect. Further, in 34.2% of the RyR2-mutant cells isoproterenol caused putative DADs and arrhythmias (Figure 6). Finally, using [Ca^2+^]_i_ imaging Fatima et al. showed that CPVT1 cardiomyocytes exhibit higher amplitudes and longer durations of spontaneous local Ca^2+^ release events already at basal state compared to the control cardiomyocytes. These findings are consistent with the irregular SR Ca^2+^ release observed in cells expressing the mutant RyR2 that underlie CPVT1.^25^

In a recent study Jung et al. reported on the generation of iPSC from a 24-year-old CPVT1 woman carrying the novel RyR2 S406L mutation.^39^ The S406L mutation is located in the N-terminal domain of the RyR2 channel, which is one of the three hot spots for CPVT-associated RyR2 mutations. Based on immunofluorescence staining of control and CPVT cardiomyocytes, the authors proposed that the S406L mutation does not interfere with trafficking of the homotetrameric channel. These authors further demonstrated that increasing the stimulation frequency was associated with a higher percentage of cells with abnormal Ca^2+^ handling in CPVT than in control cells. This frequency-induced stress is compatible with our findings^37^ that stimulation alone caused arrhythmias in CPVT but not in control cardiomyocytes. Jung et al. also reported that control and CPVT1 cardiomyocytes had similar diastolic and systolic Ca^2+^ levels, as well as comparable SR Ca^2+^ content, determined by caffeine application. However, in the presence of isoproterenol the diastolic Ca^2+^ level was significantly elevated in CPVT1 cardiomyocytes compared to control cells, while systolic Ca^2+^ remained similar; these findings are in agreement with the effects of isoproterenol on CPVT2 cardiomyocytes.^37^ Moreover, in contrast to control cardiomyocytes, SR Ca^2+^ load was not increased by isoproterenol in CPVT1 cells. Furthermore, under basal conditions, CPVT cardiomyocytes displayed abnormal Ca^2+^ sparks with a higher amplitude, prolonged (compared to control) plateau phase, longer duration at 50% peak amplitude, and longer decay time. In response to isoproterenol, in CPVT cardiomyocytes Ca^2+^ spark frequency increased compared to control cells, and the sparks had longer decay time. Finally, Jung et al. showed that in all CPVT1 cardiomyocytes stimulated with isoproterenol the DADs and triggered arrhythmias were abolished by the ryanodine antagonist dantrolene, suggesting that a defective inter-domain interaction within the RyR2 is the underlying arrhythmogenic mechanism of the S406L mutation.

## SUMMARY

CPVT is a complex disease which poses several challenges in the management of affected patients.^49^ Despite the recent advancement in understanding the diverse aspects of CPVT, this fatal disease still presents high mortality rates among young and older individuals, and therefore there is an emerging need for developing targeted pharmacological agents. Patient-specific iPSC can provide useful platforms for the discovery of unprecedented insights into disease mechanisms, as well as new drugs.^50^ Indeed, since the seminal breakthrough of generating iPSC,^31^,^51^ several patient-specific iPSC lines were developed for a variety of cardiovascular syndromes^33^–^39^ which demonstrated that their derived cardiomyocytes exhibit the electrophysiological features of the disorder, thus supporting the suitability of iPSC-derived cardiomyocytes to serve as a platform for exploring disease mechanisms in human genetic cardiac disorders. Hence, the derivation of cardiomyocytes from CPVT patients can provide the means to study, in the mutated myocytes, the functional changes and the underlying molecular mechanisms of CPVT, screen and develop candidate drugs on a patient-specific level, and thus advance our understanding of the disease and consequently improve its future clinical outcome.

Although DADs were described *in vitro* and *in vivo* in CPVT mouse models, the demonstration that these phenomena were responsible for arrhythmogenesis in humans was largely a consequence of genetic research.^52^ Therefore, our findings^37^ and those of others^38^,^39^ demonstrating the generation of DADs and triggered arrhythmias in human CPVT patient-derived cardiomyocytes are of great importance. Finally, investigating the responsiveness, to anti-arrhythmic drugs, of CPVT-mutated cardiomyocytes from individual patients may give rise to the future application of “personalized medicine,” which is likely to reduce the morbidity and mortality of patients affected by inherited arrhythmias.

## Abbreviations:

CASQ2: cardiac calsequestrin
CICR: calcium-induced calcium release
CPVT: catecholaminergic polymorphic ventricular tachycardia
CRU: calcium release unit
DADs: delayed afterdepolarizations
EB: embryoid body
E–C: excitation–contraction
ICD: intraventricular cardioverter defibrillator
iPSC: induced pluripotent stem cells
RyR2: cardiac ryanodine receptor
SR: sarcoplasmic reticulum
